# Executive Control of Emotional Conflict

**DOI:** 10.3389/fpsyg.2019.00359

**Published:** 2019-02-21

**Authors:** Ilaria Boncompagni, Maria Casagrande

**Affiliations:** ^1^Dipartimento di Psicologia, Sapienza Università di Roma, Rome, Italy; ^2^Dipartimento di Psicologia Dinamica e Clinica, Sapienza Università di Roma, Rome, Italy

**Keywords:** Attentional Network Test, alerting, orienting, executive control, emotion

## Abstract

Attentional networks and their interactions have been extensively studied through the Attentional Network Test for Interaction (ANTI). This task combines a spatial cueing paradigm with a flanker procedure and examines the efficiency and the interactions among the attentional networks (Alerting, Orienting and Executive control). However, the ANTI did not consider the effect of emotions on the attentional systems, although many studies have shown a relationship between emotion and Executive system. This study aims to analyze the executive system in an emotional context. We used a version of ANTI with arrows (ANTI-A) and an ANTI-Emotion (ANTI-E), where the arrows in the flanker task were replaced with neutral and threatening faces. One hundred and thirty-four university students performed both an ANTI-A and an ANTI-E. Results confirmed all the main effects and interactions for both the types of ANTI. Furthermore, the ANTI-E showed that the executive control of the conflict was harder when the target was neutral rather than when it was threatening. This difficulty in solving the flanker task could be due to the effect of distractors with a threatening valence. The ANTI-E could be allowed to verify how much attentional bias that characterizes people with emotional disorders (e.g., anxiety) may depend on altered executive control of the emotional conflict.

## Introduction

According to Posner and Petersen (Posner and Petersen, 1990; Petersen and Posner, 2012), the attentional system is divided into three networks: Alerting, Orienting, and Executive system. These networks are anatomically distinct and perform different functions. The Alerting system is involved in achieving (phasic alerting) and maintaining (tonic alerting) a general state of readiness of the cognitive system. The orienting system is responsible for the movement of attention through space to select and focus on the to-be-attended stimulus. The executive system allows the monitoring and resolution of the conflict between expectation, stimulus, and response.

A test that allows evaluating attentional systems simultaneously is the Attentional Network Test (ANT; Fan et al., 2002). This task is a combination of the Spatial Cueing Paradigm (Posner, 1980) and the Flanker Task (Eriksen and Eriksen, 1974). The ANT requires participants to discriminate faster and most accurate possible if the central arrow (the target) points to the right or left, ignoring the side arrows (the flankers). Before the presentation of the target and flankers, a cue is presented. The ANT includes four cue conditions: no-cue (no asterisk will appear before the presentation of the target); double-cue (two asterisks will appear: one above and one below the fixation point); spatial cue (a single asterisk will appear: above or below the fixation point) and a central cue (an asterisk will appear at the center of the screen). The flanker conditions can instead be of three types: congruent (the flankers point in the same direction as the target), incongruent (the flankers point in the opposite direction to the target) and neutral. The Alerting effect is evaluated by subtracting the RTs of the double-cue condition from the RTs of the no-cue condition. The Orienting effect is calculated by subtracting the RTs of the spatial cue (that indicated where the target is attended) from the RTs of the central cue. The Conflict effect is calculated through the subtraction of the RTs of the flanker congruent condition from the RTs of the incongruent flanker condition. A limit of this task is given by the evaluation of two attentional systems – Alerting and Orienting – through the same variable: the cue. This experimental condition does not allow independently measuring the two networks, nor their possible interactions. Another constraint of this task is due to the spatial cue that is valid in the 100% of the trials. This type of validity does not lead to assess the attentional costs given by the invalid cues. To outmatch these limits, Callejas et al. (2004, 2005) modified the original version of the ANT, inserting a new variable (a high-frequency sound) in half of the trials. This auditory warning has replaced the double-cue and has permitted to independently evaluate the Alerting system, by subtracting RTs (or accuracy) of the warning trials from RTs (or accuracy) of the no-warning trials. These authors also introduced the 50% of invalid trials. These differences make the modified version of ANT [Attentional Network Test for Interaction (ANTI)] more suitable for studying interactions between attentional networks. In particular, it showed that alerting inhibits executive control and enhances orienting (e.g., Callejas et al., 2004, 2005; Fuentes and Campoy, 2008).

Attentional networks and their interactions have been extensively studied through the use of ANT and ANTI in adults and children (e.g., Callejas et al., 2004, 2005; Rueda et al., 2004; Fuentes and Campoy, 2008; Fan et al., 2009; Poynter et al., 2010; Martella et al., 2011; Roca et al., 2011; Federico et al., 2013, 2017; Spagna et al., 2014, 2016; Luna et al., 2018) and in clinical populations (Casagrande et al., 2012, 2017; Marotta et al., 2015). These findings suggest that some manipulations of the task could allow catching other interactions among attentional networks. One relevant methodological aspect that may modulate such interactions might be the type of the stimuli (i.e., target and flankers) used to assess attentional performances. In the ANT and ANTI, arrows have been generally used to evaluate executive control. However, other types of stimuli were also used. For example, Roca et al. (2011) used machines as target and flankers stimuli. Spagna et al. (2014, 2016) used fruits or geometrical shapes (Spagna et al., 2014). Rueda et al. (2004) used fishes, while Federico et al. (2013, 2017) used schematic or real faces as stimuli.

Some studies have shown that the type of used stimuli modulated the conflict due to the flankers. For example, the use of arrows generates a more significant conflict than the use of fruit (Spagna et al., 2014), while the use of real faces looking to the left or right positively affected attentional orienting and executive control and reduced the efficiency of alerting, as compared to both schematic faces and fishes (Federico et al., 2013). This finding highlights as the social meaning of stimuli modulated the efficiency of the attentional networks. The emotional expression of a facial expression certainly has a high social value, but no study has evaluated whether the emotional valence of the stimuli affects the efficiency of the attentional networks and their interactions.

Many studies (Padmala et al., 2011; Kalanthroff et al., 2013; Allen et al., 2014; Shields et al., 2016) have shown a relationship between emotion and Executive system. Specifically, negative emotions would seem to reduce the efficiency of the Executive system. Indeed, the inhibitory control is affected by sad faces used like distractors (Gupta and Srinivasan, 2015). This result could be due to the increased arousal produced by negative emotion (Schachter and Singer, 1962; LeDoux, 1996), and it is well-known that higher is the arousal less efficient the executive functions are (Kuhbandner and Zehetleitner, 2011). Gupta et al. (2016) show that both positive and negative valence differently affect the conflict resolution. When a low perceptual load characterizes the task, distractors with both positive and negative valence interfere with the conflict solution, while distractors with a positive valence negatively affect the conflict solution when the task has a high perceptual load (Gupta et al., 2016). Another study (Srinivasan and Gupta, 2010) found that sad expressions are linked to focused attention, while happy faces are associated with the distribution of attentional resources. The emotional conflict could also affect the accuracy of the response. Indeed, if a smiling face is presented after a participant’s error, in the following trial, the conflict was perceived as higher, and it leads to a slower response. Interestingly, the slowing response did not occur when the incorrect response was followed by a sad face (Gupta and Deák, 2015).

Finally, in the resolution of emotional conflict also the hemispheric asymmetry plays a role. Right-lateralized emotional and motivational mechanisms compete for the control of non-emotional cognitive processes (Gupta and Raymond, 2012; Gupta et al., 2018).

To better understand the relationship between emotions and the executive system, Pessoa (2009) proposed the dual competition model. According to this model, the executive performance would be compromised by the presence of emotional stimuli irrelevant to the task performance. In this case, the individual cognitive resources would not be used to pursue the demands of the task, but they would also be partly allocated for the processing of the emotional load coming from irrelevant stimuli. The resources needed to resolve a conflict would be shared or removed as the consequences of the negative stimuli processing. The study of the executive system in an emotional context is of fundamental importance because it could allow defining the executive control in a conflictual emotional condition.

The present study was aimed to evaluate whether and how social stimuli (such as faces), compared to neutral stimuli (such as arrows), could affect the executive system functioning, also assessing whether the executive control is influenced by a negative emotional valence of a face. We advanced the following predictions: (a) according to previous findings (Federico et al., 2013; Spagna et al., 2014), a lower conflict should be observed when the stimuli are faces than when they are arrows, (b) the conflict should be higher when the target is a face with a neutral expression compared to when the face has a threatening expression. In fact, in the first case, the neutral value of the target extends the attentional focus on the flankers (Fenske and Eastwood, 2003) that, in the incongruent condition, have a negative valence and this latter should affect the performance. When the target stimulus is negative, the attentional focus narrows (Fenske and Eastwood, 2003), so the flankers, not falling within the attentional focus, cannot exercise their distracting effect. Furthermore, the study was aimed to evaluate whether the emotional valence of the stimuli can modulate the interaction between the executive system and the other attentional networks (i.e., Alerting and Orienting).

To assess these hypotheses we used an ANTI (Callejas et al., 2005) that employs arrows as stimuli (ANTI-A) and an Attentional Network Task for Interaction – Emotion (ANTI-E). The ANTI-E has a procedure identical to the ANTI-A with only one difference, the target and flanker stimuli (i.e., the arrows) were replaced by faces with negative or neutral emotional valence.

## Materials and Methods

### Participants

A power analysis was conducted to determine the sample size needed for the participants. The power analysis was carried out with G^∗^power software (Erdfelder et al., 1996) and revealed that a sample size of 68 participants would be adequate for detecting a significant effect and a large effect size (0.80; Cohen, 1992).

One hundred and thirty-four university students participated in the study (mean age: 23.90 ± 1.81). All participants had normal or correct vision. The experiment was conducted in line with the ethical standards of the Declaration of Helsinki, and the project was approved by the ethics committee of the Department of Psychology – Sapienza University of Rome. All participants signed informed consent.

### Apparatus

The experiment was programmed with the E-Prime software (Schneider et al., 2002) that controlled the stimuli presentation and the response recordings. Stimuli were presented on a 17 CTR monitor with a screen resolution of 1024 × 768 pixels. The participant’s responses were recorded through a standard keyboard, and the acoustic tone was presented through headphones (Quasar Headset, Trust.com).

### ANTI-A

#### Stimuli

Each trial began with the presentation of a central cross of 1° (degrees of visual angle) on a gray background. The stimuli consisted of a row of five black arrows. The target was a right- or a left-pointing arrow at the center, which was flanked on both sides by two arrows pointing either in the same direction (congruent trials) or the opposite direction (incongruent trials). A single arrow consisted of 0.58°, and the contours of adjacent arrows were divided by 0.06°. The stimuli (the central arrow plus four flankers) subtended a total of 3.27°. According to Callejas et al. (2004, 2005), the target and flankers were presented 1.06° above or below the fixation point. The cue was an asterisk of 1°, and it was presented at the position of the upcoming target (valid cue condition), in the opposite location (invalid cue condition), or it could be absent (no-cue condition). All stimuli were black and were presented on a gray background [RGB (160, 160, 160), 31.25 cd/m^2^] maintaining luminance approximately constant between stimuli and background (3.35–6.25 cd/m^2^). The auditory warning stimulus was a 98-dB and 2000 Hz sound, lasting 50 ms (Martella et al., 2014).

#### Procedure

**FIGURE 1:**
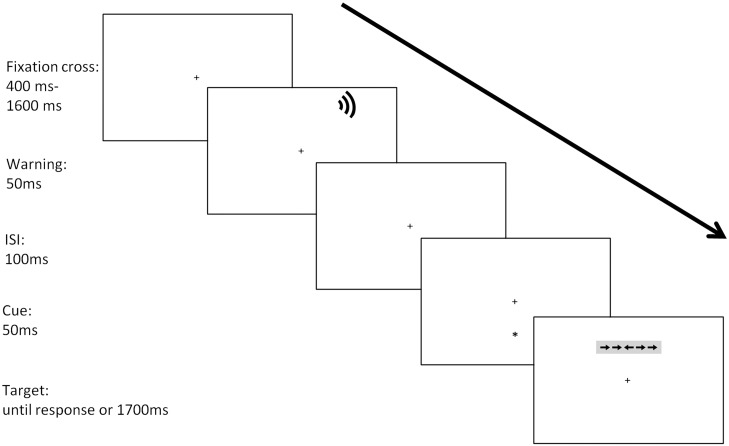
Stimuli and sequence of events in the ANTI-A.

Participants were individually tested in a silent and dimly illuminated (approximately 20 lux) room, at a 50 cm distance from the computer screen. Each trial began with a fixation cross period of variable duration (400–1600 ms). In half of the trials, the fixation cross was followed by the warning stimulus lasting 50 ms. Next, a cue lasting 150 ms was presented. An asterisk appeared in the same position of the upcoming target (valid condition: 33% of the trials), or the opposite position than the one signaled by the cue (invalid condition; 33% of the trials); in the no-cue condition, no orienting stimulus was presented. After a fixed interstimulus interval (ISI) of 350 ms, the target was presented for 150 ms and participants had a limit of 1700 ms to respond. The fixation point was at the center of the screen throughout the trial. Participants are required to keep their gaze on the fixation cross and respond as quickly and accurately as possible to the target stimulus. The sequence of the experimental procedure for each trial is shown in Figure 1.

### ANTI-E

#### Stimuli

Four threatening faces and four neutral faces were selected from the database used by Maccari et al. (2014). All pictures measured 890 × 1300 pixels (2 cm × 3 cm) with a resolution of 72 dpi on the screen. The faces have been balanced by gender, and the gaze direction was straight.

#### Procedure

**FIGURE 2:**
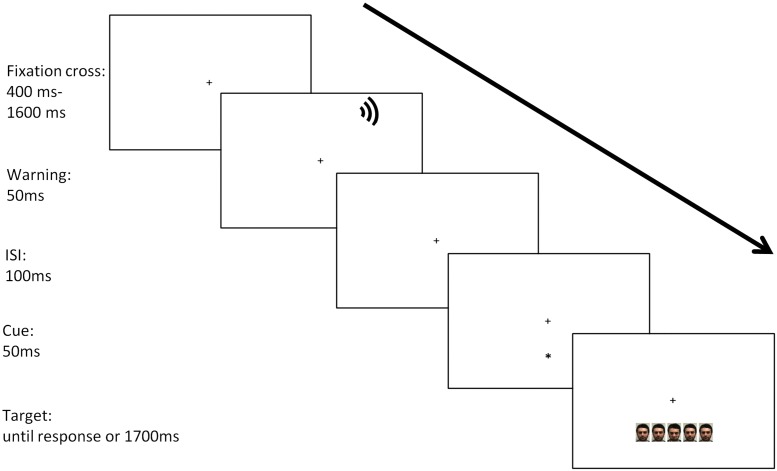
Stimuli and sequence of events in the ANTI-E.

The procedure was the same as described for the ANTI. The only variation concerns the stimuli. In the ANTI-E the target and the flankers were faces. The faces have two values: neutral and threatening. Participants are asked to keep their eyes on the fixation point and to respond as quickly and accurately as possible to the target. The task involves discriminating the emotion of the face centrally presented by pressing one of two keys on the keyboard. The keys have been balanced between subjects. The stimuli and the sequence of events are shown in Figure 2.

### General Procedure

Half of the participants were required to respond by pressing the right button of the mouse when the arrow pointed to right or when the face had a neutral expression and by clicking the left button when the arrow pointed to left or when the face had a threatening expression; for the other half of participants, response buttons were inverted. The order of ANTI and ANTI-E was balanced between subjects.

### Data Analysis

According to a consolidated ANT analysis, only the RTs of correct responses ranging between 200 and 1200 ms were considered (Callejas et al., 2005; Spagna et al., 2014).

For the ANTI-A, a Warning (Warning, No-warning) × Cue (Valid, Invalid, No-cue) × Flanker (Congruent, Incongruent) repeated measures analysis of variance (ANOVA) was conducted on both RTs of the correct responses and accuracy. For the ANTI-E, an Emotion (Neutral, Threatening) × Warning (Warning, No-warning) × Cue (Valid, Invalid, No-cue) × Flanker (Congruent, Incongruent) repeated measures ANOVA was conducted on RTs of the correct responses. The same ANOVA design was used to analyze the number of correct responses.

In both the tasks, the Warning condition was used to evaluate the Alerting effect (RTs no-warning - RTs warning), the Cue evaluated the Orienting effect (RTs invalid-cue - RTs valid-cue), Flanker trials provided a measure of Executive conflict control effect (RTs incongruent trials - RTs congruent trials); in the ANT-E, the Emotion was used to assess emotional valence.

To estimate the efficiency of each attentional system, that enable us to compare the results of this study with previous studies using the ANTI (e.g., Callejas et al., 2005; Spagna et al., 2014) a one-way ANOVA considering the Task was performed on the Alerting, the Orienting, the Executive Control Conflict effects.

A high score of the orienting effect reflects the ability to rapidly orient the attention to the targets appearing in the cued positions. A smaller conflict effect reflects the participant’s ability to inhibit the interfering effect brought by distractor stimuli (the flankers). The alerting effect represents the benefit of alerting on the speed of the response to the target.

To directly compare the effects of the two types of stimuli on the attentional effects a one-way ANOVA considering the Type of the target stimulus (Threatening Face, Neutral Face, Arrow) was made on Alerting, Orienting and Executive Attentional Control.

To analyze the main effects and the interactions panned comparisons have been used.

An α value of 0.05 was used to establish statistical significance for all analyses.

## Results

### ANTI-A

#### Reaction Times

Table 1 shows the means RTs (± SD) for each experimental condition. All main effects were significant: Warning [*F*(1,133) = 404.56; *p* < 0.0001; η^2^ = 0.75], Cue [*F*(2,266) = 691.33; *p* = 0.0001; η^2^ = 0.84], and Flanker [*F*(1,133) = 1160.93; *p* < 0.0001; η^2^ = 0.90]. Specifically, the TRs were faster in the Warning than in the No-Warning Condition (582.83 vs. 604.62 ms); the TRs were faster in the Valid than in the Invalid trials (545.98 vs. 622.10 ms; *F*(1,33) = 787,35; *p* < 0.0001; η^2^ = 0.10] and for the Valid trials compared to the No Cue trials [545.98 vs. 613.10 ms; *F*(1,133) = 821.94; *p* < 0.0001; η^2^ = 0.12]; the TRs were faster in the Congruent than Incongruent trials (557.47 vs. 629.98 ms). The Warning × Cue [*F*(2,266) = 12.01; *p* < 0001; η^2^ = 0.35] interaction revealed faster RTs in the valid than invalid trials in the Warning condition compared to the No-Warning condition [the difference between valid and invalid trials was 79.60 ms in the Warning condition; *F*(1,133) = 638.88; *p* < 0.0001; η^2^ = 0.84; while it was 72.64 ms in the No-Warning trials; *F*(1,133) = 656.86; *p* < 0.0001; η^2^ = 0.83]. The Warning × Flanker interaction [*F*(1,133) = 70.60; *p* < 0.0001; η^2^ = 0.35] showed a higher conflict in the presence of the Warning, compared to the No-Warning condition [81.46 ms, *F*(1,133) = 947.33; *p* < 0.0001; η^2^ = 0.88; 63.57 ms, *F*(1,133) = 935.14; *p* < 0001; η^2^ = 0.88, respectively]. The Cue × Flanker interaction [*F*(2,266) = 3.10; *p* < 0.05; η^2^ = 0.02] indicated a higher executive control of conflict when the trials were valid (68.52 ms), compared to both invalid [74.09 ms, *F*(1,133) = 4.69; *p* = 0.03; η^2^ = 0.03] and No-Cue conditions [104.93 ms; *F*(1,133) = 4.26; *p* = 0.04; η^2^ = 0.03].

**Table 1:** Means ( ± SD) of reaction times and percentage of correct responses for each experimental condition in the ANTI-A.

		No warning			Warning		
		No cue	Invalid	Valid	No cue	Invalid	Valid
Reaction times	Congruent	595.33 (67.08)	595.97 (66.55)	527.19 (69.16)	555.94 (64.93)	574.14 (66.29)	496.24 (69.07)
	Incongruent	658.31 (72.33)	663.69 (74.15)	587.19 (75.81)	642.82 (75.54)	654.59 (73.45)	573.28 (79.04)
Accuracy	Congruent	97.43 (0.47)	97.88 (0.46)	98.48 (0.35)	98.37 (0.41)	98.58 (0.38)	98.97 (0.36)
	Incongruent	93.35 (0.65)	93.47 (0.83)	95.40 (0.38)	94.43 (0.62)	93.40 (0.89)	94.66 (0.68)


#### Accuracy

The accuracy was 96%. The main effects of Warning [*F*(1,133) = 6.90; *p* < 0.01; η^2^ = 0.05], Cue [*F*(2,266) = 9.90; *p* < 0.001; η^2^ = 0.07], and Flanker [*F*(1,133) = 99.63; *p* < 0.0001; η^2^ = 0.43] were significant. The accuracy was higher in the Warning compared to No-Warning trials (96.44 vs. 96.00), in the valid compared to both Invalid trials [96.88 vs. 95.84; *F*(1,133) = 13.59; *p* < 0.0001; η^2^ = 0.09] and No-Cue trials [96.88 vs. 95.97; *F*(1,133) = 17.63; *p* < 0.0001; η^2^ = 0.12], and in the Congruent than Incongruent trials (98.34 vs. 94.13). The Warning × Cue [*F*(2,266) = 5.47; *p* < 001; η^2^ = 0.04] interaction showed higher accuracy in the valid than invalid trials in the Warning condition compared to the No-Warning condition [the difference between valid and invalid trials was -0.84 in the Warning condition; *F*(1,133) = 4.99; *p* = 0.02; η^2^ = 0.04; while it was -1.25 in the No-Warning trials; *F*(1,133) = 16.19; *p* < 0.0001; η^2^ = 0.11]. The Warning × Flanker [*F*(1,133) = 6.48; *p* = 0.01; η^2^ = 0.05] interaction was significant and indicated a higher accuracy in the presence of the Warning, compared to the No-Warning condition, only in the Congruent trials [99.84 vs. 97.94; *F*(1,133) = 20.59; *p* < 0.0001; η^2^ = 0.13].

### ANTI-E

#### Reaction Times

Table 2 shows the mean RTs ( ± SD) for each experimental condition. All the main effects were significant: Warning [*F*(1,133) = 33.71; *p* < 0.0001; η^2^ = 0.20]; Cue [*F*(2,266) = 358.85; *p* < 0.0001; η^2^ = 0.73]; Flanker [*F*(1,133) = 5.08; *p* < 0.02; η^2^ = 0.04]; Emotion [*F*(1,133) = 87.34; *p* < 0.0001; η^2^ = 0.40]. The RTs were faster in the Warning than in the No-warning trials (787.70 vs. 798.83 ms). RTs were also faster in the valid compared to both Invalid trials [759.16 vs. 807.37 ms; *F*(1,133) = 408.26; *p* < 0.0001; η^2^ = 0.99] and No-Cue trials [759.16 vs. 811.75 ms; *F*(1,133) = 516.63; *p* < 0.0001; η^2^= 0.80]. RTs were faster in the congruent than incongruent trials (790.72 vs. 794.80 ms). Finally, RTs were faster when the target was a threatening face compared to when it was a neutral face (776.40 vs. 809.12 ms). The Warning × Cue interaction [*F*(2,266) = 12.27; *p* < 0001; η^2^ = 0.08] revealed faster RTs in the Valid then invalid trials in the Warning condition [55.28 ms; *F*(1,133) = 311.64; *p* < 0.0001; η^2^ = 0.70] compared to the No-Warning condition [41.19 ms; *F*(1,133) = 259.44; *p* < 0.0001; η^2^ = 0.66]. The Cue × Flanker interaction [*F*(2,266) = 4.02; *p* < 0.02; η^2^ = 0.02] indicated a higher executive control of conflict in the valid trials (Executive Control 0.15 ms), compared to No-Cue trials [Executive Control 9.87 ms; *F*(1,133) = 7.02; *p* < 0.001; η^2^ = 0.05]. The Cue × Emotion interaction [*F*(2,266) = 17.05; *p* < 0.0001; η^2^ = 0.12] revealed faster responses to the negative emotion in the Valid trials, compared to the Invalid trials [736.97 vs. 793.11 ms; *F*(1,133) = 330.27; *p* < 0.0001; η^2^ = 0.71] and No Cue trials [736.97 vs. 799.12 ms; *F*(1,133) = 511.45; *p* < 0.0001; η^2^ = 0.79]. The critical interaction Flanker × Emotion was significant [*F*(1,133) = 5.20; *p* = 0.02; η^2^ = 0.04]. Planned comparisons showed that the RTs were faster in the congruent trials, compared the incongruent trials, only in the neutral emotion [805.21 vs. 813.03 ms; *F*(1,133) = 8.95; *p* < 0.001; η^2^ = 0.06].

**Table 2:** Means ( ± SD) of reaction times and percentage of correct responses for each experimental condition in the ANTI-E.

		Neutral						Threatening					
		No warning			Warning			No warning			Warning		
		No cue	Invalid	Valid	No cue	Invalid	Valid	No cue	Invalid	Valid	No cue	Invalid	Valid
**Reaction times**	Congruent	824.37 (77.54)	823.08 (78.80)	784.78 (90.16)	811.82 (83.54)	816.93 (85.35)	770.30 (89.92)	801.35 (76.40)	792.59 (77.62)	751.09 (91.41)	789.72 (79.28)	791.90 (81.16)	730.76 (88.30)
	Incongruent	837.96 (76.96)	829.26 (81.60)	790.12 (88.24)	823.37 (85.01)	827.31 (79.29)	780.18 (84.23)	812.32 (80.12)	796.39 (78.32)	740.55 (77.85)	793.10 (74.97)	791.87 (79.48)	725.47 (78.25)
**Accuracy**	Congruent	80.55 (1.45)	79.29 (1.52)	82.09 (1.34)	81.30 (1.43)	81.25 (1.40)	83.12 (1.24)	75.56 (1.15)	77.28 (1.35)	77.75 (1.30)	77.14 (1.32)	75.87 (1.39)	78.68 (1.28)
	Incongruent	80.32 (1.39)	79.10 (1.41)	81.81 (1.44)	78.68 (1.56)	78.87 (1.46)	81.44 (1.42)	77.19 (1.42)	76.31 (1.39)	78.59 (1.28)	78.87 (1.27)	78.54 (1.34)	79.48 (1.33)


#### Accuracy

The accuracy was 79%. The main effects of Cue [*F*(2,266) = 18.64; *p* < 0.0001; η^2^ = 0.12] and Emotion [*F*(1,133) = 7.33; *p* < 0.007; η^2^ = 0.05] were significant. The accuracy was higher in the valid compared to both Invalid trials [80.38 vs. 78.69; *F*(1,133) = 33.24; *p* < 0.0001; η^2^ = 0.20] and No Cue trials [80.38 vs. 78.31; *F*(1,133) = 21.05; *p* < 0.0001; η^2^ = 0.14] and the accuracy was higher in response to neutral than threatening stimuli (80.63 vs. 77.63). Further, the Flanker × Emotion interaction [*F*(1,133) = 12.18, *p* < 0.001; η^2^ = 0.08] revealed a higher accuracy in the congruent trials, compared to incongruent trials only in the Neutral condition [81.25 vs. 80.00; *F*(1,133) = 5.61, *p* < 0.01; η^2^ = 0.04].

#### Attentional Effects

**FIGURE 3:**
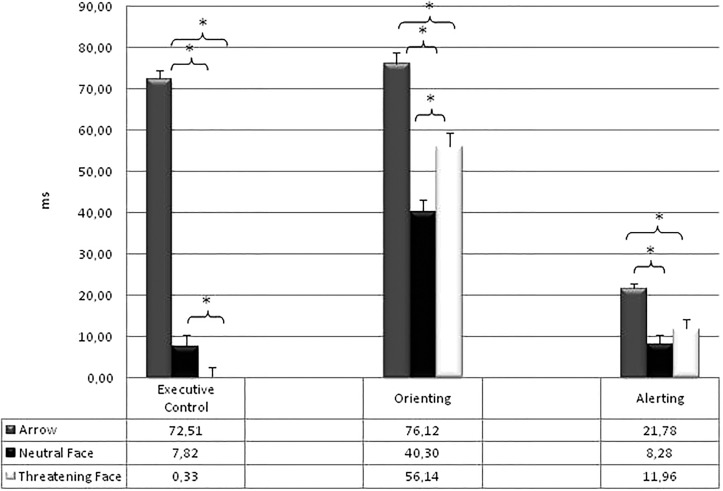
Mean RTs (and standard error) of the attentional effects for each type of stimulus. ^∗^*p* < 0.05.

Table 3 shows the mean RTs ( ± SD) of the attentional effects for each type of stimulus. All the attentional effects resulted significant: Alerting [*F*(2,266) = 16,32; *p* < 0.0001; η^2^ = 0.11]; Orienting [*F*(2,266) = 56.31; *p* < 0.0001; η^2^ = 0.30]; Executive Control of Conflict [*F*(2,266) = 317.23; *p* < 0.0001; η^2^ = 0.70]. The Warning effect was higher in the Arrows condition (21.78 ms) compared to both the Neutral [8.28 ms; *F*(1,133) = 35.24; *p* < 0.0001; η^2^ = 0.21] and the Threatening conditions [11.96 ms; *F*(1,133) = 16.64; *p* < 0.0001; η^2^ = 0.11]. The Orienting effect was higher in the Arrows condition (76.12 ms) with respect to both the Neutral [40.30 ms; *F*(1,133) = 130.00; *p* < 0.0001; η^2^ = 0.49] and the Threatening conditions [56.14 ms; *F*(1,133) = 33.06; *p* < 0.0001; η^2^ = 0.20]; also the Orienting effect was higher in the threatening than in the Neutral condition [*F*(1,133) = 20.26; *p* < 0.0001; η^2^ = 0.13]. Finally, the Conflict effect was higher in the Arrows condition (72.51 ms) with respect to both the Neutral [7.82 ms; *F*(1,133) = 453.17; *p* < 0.0001; η^2^ = 0.77] and the Threatening conditions [0.33 ms; *F*(1,133) = 532.68; *p* < 0.0001; η^2^ = 0.80]. Furthermore a higher difficulty in resolving the conflict was present in the Neutral than in the Threatening condition [*F*(1,133) = 5.20; *p* < 0.02; η^2^ = 0.14]. The Figure 3 reports the attentional effects for the three types of target stimuli.

**Table 3:** Means ( ± SD) for each type of target stimulus of the in the two versions of the ANTI.

	Arrow	Neutral face	Threatening face
Alerting	21.78 (12.54)	8.28 (24.62)	11.96 (25.95)
Orienting	76.12 (31.40)	40.30 (32.83)	56.14 (35.76)
Executive control	72.51 (24.54)	7.82 (30.25)	0.33 (26.14)


## Discussion

The primary aim of this study was to compare executive conflict control when neutral and emotional stimuli are used. To test this hypothesis, a new version of the ANTI was developed, modifying the flanker task. In particular, the arrows have been replaced with faces that had negative or neutral emotional expression.

For both the ANTI-A and the ANTI-E, the results confirm all the main effects and interactions (e.g., Callejas et al., 2005; Spagna et al., 2014). Overall, the pattern of results obtained shows that the new version of the ANTI, proposed in this study, reliably assess each attentional system, i.e., alerting, orienting and executive systems, underlining the high validity of the Attentional Network Test.

According to Spagna et al. (2014), our findings show a higher conflict when the arrows were used as stimuli compared to when the stimuli were faces. However, this result was observed despite the higher difficulty in the execution of the ANTI-E compared to the ANTI-A, as indicated by a lower level of accuracy. The worse accuracy of the ANTI-E, compared to the ANTI-A, is general and not specific to a given condition (e.g., flanker, cue, or warning) and it may depend on the higher complexity required to process a face compared to that needed to process an arrow.

The most compelling result is observed in the different Conflict effect when the target was a neutral than a threatening face. Specifically, the executive control of the conflict was more difficult when the target was neutral rather than when it was threatening. This finding would seem to confirm the hypothesis of Pessoa (2009). The difficulty in solving the flanker task when the target was a neutral face could be due to the effect of distractors with a threatening valence. The participant’s performance would be more affected by the negative emotion of the faces. In this case, the resources for the resolution of the conflict would be diverted from the negative emotion, which would result in a delayed response.

According to Fenske and Eastwood (2003), would produce a restriction of attentional focus, making ineffective the distractor effect of incongruent flankers. Conversely, the authors hypothesize a widening of the attentional focus in the presence of targets with positive emotional valence. This different allocation of attentional resources could explain the higher conflict present when the target was neutral rather than threatening. When the target stimulus was a threatening face, the conflict was virtually absent (2 ms). The narrowing of the attention focus has set the flankers adjacent to the target out of the attentional focus itself and in this way their distracting effect has been annulled.

Also, the Broaden-and-Build theory of positive emotions (Fredrickson, 2004) agrees with these conclusions. This theory proposes that both positive and negative emotions affect the attentional resources. The positive emotions would broaden the thoughts, the actions, and attentional resources, leading to long-term benefits and an increase of personal resources. Conversely, the negative emotions would allocate the attentional resources quickly for the survival. Several studies (Srinivasan and Gupta, 2010, 2011; Srivastava and Srinivasan, 2010) confirm this theory and found an increase of attentional spotlight when happy faces are presented.

The analysis of accuracy has indicated a higher number of correct answers for the neutral, rather than the threatening faces. This result could suggest that the resolution of the conflict would be harder in an emotional than the neutral condition. This finding confirms previous studies (Egner and Hirsch, 2005; Zinchenko et al., 2015) that reported a higher number of errors in emotionally salient conditions. Furthermore, this result is also consistent with Srinivasan and Gupta (2010, Experiment 2) findings. These authors observed that when attentional resources are reduced, and negative faces were used as distractors, the participants made many errors.

In general, the results of the present study further confirm that ANTI is a reliable test in the study of attentional networks. In conclusion, it can be observed that, although the three attentional networks are anatomically independent (Posner and Dehaene, 1994), various interactions between the attentional systems can be demonstrated (Callejas et al., 2005; Casagrande et al., 2012; Spagna et al., 2016). These interactions are confirmed even when the test requires emotional processing and not only when neutral stimuli are used.

The principal limitation of the present study could be the lack of the evaluation of the emotional state of participants that could have indicated how the latter can modulate the “cold” and “hot” components of the executive control of the conflict; this aspect should be assessed in future studies. The results observed with the ANTI-E adds new and interesting information concerning the way in which attentional systems work and interact to reach an adaptive behavior in front of the different kinds of stimuli. This emotional version of the ANTI could open to the possibility to use this test in the studies of the executive control of emotional conflict in people with altered emotional regulation, such as in anxious or depressed people, or people with high trait anxiety. In fact, in the study of attentional bias in trait anxiety (e.g., Fox et al., 2002; Koster et al., 2006; Mogg et al., 2008) has been reported the relevant role of attentional control (Derryberry and Reed, 2002; Pacheco-Unguetti et al., 2010), but attentional control has been assessed considering only the “cold” components of the executive system. In psychological disorders, such as anxiety or depression, or other behavioral diseases it might be useful to be able to evaluate also the “hot” components of the executive system. The results of the present study support this opportunity.

## Author Contributions

IB and MC designed the experiments, analyzed the data, and wrote the manuscript. IB has administered the experiments.

## Conflict of Interest Statement

The authors declare that the research was conducted in the absence of any commercial or financial relationships that could be construed as a potential conflict of interest.
